# Erratum

**DOI:** 10.5935/abc.20190170

**Published:** 2019-08

**March issue of 2019, vol. 112(3), pages 326-368**

In the “The Brazilian Society of Cardiology and Brazilian Society of Exercise and Sports Medicine Updated Guidelines for Sports and Exercise Cardiology - 2019”, the following names have been omitted from the publication: Japy Angelini Oliveira Filho, associate editor and fifth name in the authorship, from the Universidade Federal de São Paulo (UNIFESP); Antonio Claudio Lucas da Nobrega, from the Universidade Federal Fluminense; Luiz Gustavo Marin Emed, from the Hospital Cardiológico Costantini; and Roberto Vital, from the Comitê Paralímpico Brasileiro (CPB) and Universidade Federal do Rio Grande Do Norte (UFRN), inserted at the end of the authorship, in this order, and their respective institutions.

**June issue of 2019, vol. 112(6), pages 775-781**

In original article “The Olympic Experimental Gymnasium Program and its Association with the Prevalence of Cardiovascular Risk Factors in Adolescents: A Cross-Sectional Study”, consider Carlos Scherr as the correct form for the name of the author Carlos Scheer.

**July issue of 2019, vol. 113(1), pages 20-30**

In the original article “Aplicação de Escores de Risco em Síndromes Coronárias Agudas: Como o ProACS se Comporta Diante de Outros Escores de Risco?” Correct the title of Figure 1 of Cruva ROC - Escores de Risco e mortalidade hospitalar for Curva ROC - Escores de Risco e mortalidade hospitalar.

**July issue of 2019, vol. 113(1), pages 62-68**

In figure 1 of the original article “The Profile of the Brazilian Cardiologist - A Sample of Members of the Brazilian Society of Cardiology” there is an error for the category of women with 0 children. Instead of 4.5% consider correct 45%.

